# Demographics and Practice Attributes of Physician Assistants in Neurology

**DOI:** 10.1001/jamanetworkopen.2024.58839

**Published:** 2025-02-11

**Authors:** Mirela Bruza-Augatis, Nicholas M. Hudak, Roderick S. Hooker, Kasey Puckett, Andrzej Kozikowski

**Affiliations:** 1 National Commission on Certification of Physician Assistants, Johns Creek, Georgia; 2 Seton Hall University–Interprofessional Health Sciences Campus, Nutley, New Jersey; 3 Duke University School of Medicine, Durham, North Carolina; 4 Independent researcher, Ridgefield, Washington

## Abstract

This cohort study examined demographic and practice characteristics of physician assistants in neurology compared with those in all other specialties.

## Introduction

A supply and demand model indicates a shortage of neurologists by 2025,^1^ highlighting the need for more physician assistants (PAs) and nurse practitioners in this specialty.^2^ However, research on PAs in neurology is lacking. This study examines the demographics and practice attributes of PAs in neurology compared with PAs in all other medical disciplines.

## Methods

The Sterling Institutional Review Board deemed this study exempt from review and informed consent because it does not constitute human participants research. We conducted a cross-sectional study using the 2022 National Commission on Certification of PAs dataset (response rate, 83.6%) and followed the Strengthening the Reporting of Observational Studies in Epidemiology (STROBE) reporting guideline. This dataset included information on detailed demographics, practice attributes, and essential workforce information for 117 748 board-certified PAs (eMethods in Supplement 1). We conducted descriptive and bivariate analyses (χ^2^ for categorical data or Mann-Whitney *U* tests for continuous data) of workforce characteristics, comparing 1215 PAs in neurology with 116 533 PAs in all other disciplines using SPSS software version 29.0 (IBM) with statistical significance set at 2-sided *P* < .05. Data were analyzed from June to August 2023.

## Results

Among 117 748 board-certified PAs, we found that 1215 (1.0%) PAs self-identified as practicing in neurology as of 2022. PAs in neurology, compared with PAs in all other specialties, were younger (median [IQR] age, 36 [31-45] vs 39 [33-48] years) and were more likely to identify as female (81.4% vs 69.5%) and reside in an urban location (96.0% vs 92.5%) and in the Northeast (32.0% vs 24.5%) (all *P* < .001). PAs in neurology vs their colleagues in all other specialties were more likely to be hospital based (54.5% vs 41.5%). PAs in neurology were certified for fewer years (median [IQR], 8 [4-15] vs 10 [5-18] years) and worked primarily in 1 clinical position (89.2% vs 84.9%) (all *P* < .001). Compared with PAs across other specialties, almost one-third of PAs in neurology worked more than 40 hours per week (30.3% vs 29.4%; *P* < .001), and more than half reported providing telemedicine services (54.9% vs 40.1%; *P* < .001). PAs in neurology report lower compensation (median [IQR] income, $105 000 [$95 000-$125 000] vs $115 000 [$105 000-$135 000]; *P* < .001) (Table). Comparable with their PA colleagues in all other disciplines, PAs in neurology indicated similar levels of job satisfaction (83.5% vs 83.6%; *P* = .95) and burnout symptoms (31.8% vs 32.2%; *P* = .75) (Figure). PAs in neurology were less likely to plan for retirement in the next 5 years than those in all other specialties (4.2% vs 5.8%; *P* = .02).

**Table.** Demographic and Practice Characteristics of PAs in Neurology Compared With PAs in All Other Specialties

Characteristic	PAs, No. (%)		*P* value^a^
	Neurology (n = 1215)	All other specialties (n = 116 533)	
**Demographics**			
Age, y			
Mean (SD)	39.3 (10.6)	41.3 (10.9)	<.001
Median (IQR)	36 (31-45)	39 (33-48)	
Gender			
Female	989 (81.4)	81 021 (69.5)	<.001
Male	226 (18.6)	35 498 (30.5)	
Race			
Asian	68 (5.8)	7053 (6.3)	.41
Black or African American	39 (3.3)	3840 (3.4)	
White	1005 (86.1)	94 192 (84.4)	
Multiple race	25 (2.1)	2526 (2.3)	
Other^b^	30 (2.6)	3935 (3.5)	
Ethnicity			
Hispanic or Latino	48 (4.1)	7620 (6.8)	<.001
Non-Hispanic or non-Latino	1122 (95.9)	104 476 (93.2)	
Urban-rural location			
Urban	1164 (96.0)	107 204 (92.5)	<.001
Rural or isolated	48 (4.0)	8673 (7.5)	
**Practice-level **			
Years certified as a PA			
Mean (SD)	10.5 (8.0)	12.2 (8.8)	<.001
Median (IQR)	8 (4-15)	10 (5-18)	
Hours worked weekly			
≤30	109 (9.0)	15 655 (13.4)	<.001
31-40	738 (60.7)	66 534 (57.1)	
41-50	307 (25.3)	27 029 (23.2)	
≥51	61 (5.0)	7259 (6.2)	
Income, median (IQR), $	105 000 (95 000-125 000)	115 000 (105 000-135 000)	<.001
Participation in telemedicine			
No	547 (45.1)	69 535 (59.9)	<.001
Yes	667 (54.9)	46 570 (40.1)	

Abbreviation: PAs, physician assistants or associates.

^a^ Calculated using χ^2^ for categorical data or Mann-Whitney *U* tests for continuous data.

^b^ Includes PAs who selected American Indian or Alaska Native, Native Hawaiian or Pacific Islander, and other.

**Figure.**
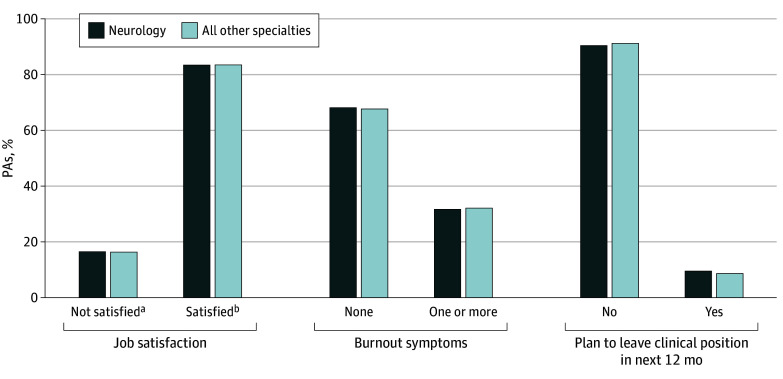
Other Significant Characteristics of Physician Assistants (PAs) in Neurology Compared With PAs in All Other Medical Disciplines ^a^Not satisfied includes neither satisfied nor dissatisfied, somewhat dissatisfied, mostly dissatisfied, and completely dissatisfied. ^b^Satisfied includes completely satisfied, mostly satisfied, and somewhat satisfied.

## Discussion

This cohort study is the first national analysis of the demographic and practice characteristics of PAs practicing in neurology, to our knowledge. Over the past decade, the number of PAs in neurology increased 170%, from 450 in 2013 to 1215 in 2022.^3^ Meanwhile, neurologists increased by 11.2% (13 156 in 2013 to 14 636 in 2022) during the same period.^4^ In terms of demographics, the median age of neurology PAs was younger than that of all other specialties, which has some implications for employment tenure. Most PAs in neurology self-identified as female (81.4%), while neurologists tend to be predominantly male (62.7%).^4^ Moreover, a smaller proportion (31.8%) of PAs in neurology reported symptoms of burnout compared with 46% of neurologists in 2022.^5^ Lower symptoms of burnout have been correlated with job and career satisfaction in various medical professions and specialties, suggesting that PAs in neurology may be more likely to continue in their current field.

We also found that 54.9% of PAs in neurology provided telemedicine services for their patients. Some view telemedicine as enhancing health care and patient safety in the postpandemic period.^6^ While there is strong evidence of telemedicine effectiveness in stroke management, the care of patients with other neurological disorders by telemedicine needs further investigation. Knowing the census of clinicians is a key step in maintaining a current understanding of how neurology care is delivered. A limitation of this study is the reliance on self-reported data. Future research should investigate the cost-effectiveness and scope of practice for PAs in neurology.

## Notes

Supplement 1.
eMethods.

Supplement 2.
Data Sharing Statement
