# Supplementary material for: Demographics and Practice Attributes of Physician Assistants in Neurology
Source: JAMA Netw Open. 2025 Feb 11;8(2):e2458839. doi: 10.1001/jamanetworkopen.2024.58839 (PMC11815520; doi:10.1001/jamanetworkopen.2024.58839)
Supplement: Supplement 1. — eMethods. [file jamanetwopen-e2458839-s001.pdf]

Supplemental Online Content

Bruza-Augatis M, Hudak NM, Hooker RS, Puckett K, Kozikowski A. Demographics and practice attributes of physician assistants in neurology. *JAMA Netw Open*. 2025;8(2):e2458839. doi:10.1001/jamanetworkopen.2024.58839

eMethods

This supplemental material has been provided by the authors to give readers additional information about their work.

© 2025 Bruza-Augatis M et al. *JAMA Network Open*.

eMethods.

Demographic characteristics include age, participants' self-reported gender (male vs female), self-reported race [American Indian or Alaska Native, Asian, Black or African American, Native Hawaiian or Other Pacific Islander, or White], and ethnicity [Hispanic or Latino and Hispanic or Latino], urban-rural location (urban vs rural/isolated), US region (Northeast, Northwest, South West), and postgraduate fellowship/residency (yes vs no).

Practice attributes include practice setting, years certified (continuous), patients seen per week (continuous), hours worked per week (continuous), and participation in telemedicine (yes vs no).

Other essential workforce information include income, job satisfaction (7-point scale from completely unsatisfied to completely satisfied which was dichotomized to satisfied vs not satisfied), burnout (5-point scale from no symptoms of burnout to complete burnout, which was dichotomized to no burnout vs one or more symptoms or burnout), intent to leave the principal clinical position within the next 12 months (yes vs no).

© 2025 Bruza-Augatis M et al. *JAMA Network Open*.
