# Supplementary material for: Demographics and Practice Attributes of Physician Assistants in Neurology
Source: JAMA Netw Open. 2025 Feb 11;8(2):e2458839. doi: 10.1001/jamanetworkopen.2024.58839 (PMC11815520; doi:10.1001/jamanetworkopen.2024.58839)
Supplement: Supplement 2. — Data Sharing Statement [file jamanetwopen-e2458839-s002.pdf]

Data Sharing Statement

Bruza-Augatis. Demographics and Practice Attributes of Physician Assistants in Neurology. *JAMA Netw Open*. Published February 11, 2025. doi:10.1001/jamanetworkopen.2024.58839

Data
Data available: No

Additional Information
Explanation for why data not available: Some or all datasets generated during and/or analyzed during the current study are not publicly available but are available from the corresponding author on reasonable request.
